# A comprehensive review of summer mortality syndrome in fish: Causes, climate impact, and mitigation strategies

**DOI:** 10.5455/javar.2025.l951

**Published:** 2025-09-12

**Authors:** Manal E. Shafi

**Affiliations:** Sustainable Agriculture Production Research Group, Department of Biological Sciences, Zoology, King Abdulaziz University, Jeddah, Saudi Arabia

**Keywords:** Fish health, mortality, pathogens, climate change, aquaculture management, herbal additives

## Abstract

Global warming poses a significant threat to aquatic animals, particularly fish production, because of the rising summer temperatures, leading to issues such as summer mortality syndrome. This issue could impact fish farms, leading to 30%–100% mortality rates and significant financial losses. This study aims to identify the underlying causes of summer mortality syndrome in fish and to evaluate modern mitigation strategies to protect aquaculture systems effectively. Mortality rates of stocked fish ranged between 30% and 70%, with some instances reaching 100%. The reasons behind summer mortality syndrome are complex and multifactorial. Contributing factors include pathogenic agents such as bacteria, viruses, fungi, and parasites, as well as poor management practices and unfavorable environmental conditions. Most types of bacteria that caused mortality rates in many fish species were *Aeromonas *spp., *Flexibacter columnaris*, *Flavobacterium columnare*, *Pseudomonas *spp., and Enterobacteriaceae, namely* Edwardsiella tarda*, *Yersinia *spp., and *Streptococcus *spp. To mitigate the impact of this syndrome, several protective measures can be implemented. These include applying some nutritional inventions such as herbal additives, essential oils, natural compounds, water management, antibiotics, vaccinations, modern technology, and improved management practices. By addressing these factors comprehensively, the risk of summer mortality syndrome can be significantly reduced. This review provides further evidence regarding the effects of summer mortality syndrome in fish. The main causes of this phenomenon are pathogens and poor management. Nutritional additives and vaccinations were effective ways to mitigate these harmful effects to maintain fish production. Further research is needed to evaluate the long-term effectiveness of these mitigation strategies and to develop guidelines for sustainable fish farming practices under changing climatic conditions.

## Introduction

In addition to being a significant food source for humans, fish is also highly valuable economically [1–5]. Profitability and human nutritional health depend on it [6]. Fish disease is one of the challenging issues impeding the global expansion of tilapia aquaculture [7]. Fish infections usually result from a combination of factors [8]. Low dissolved oxygen (L-DO) induced by water temperatures impedes the immune system and raises the susceptibility of fish to secondary bacterial infections when combined with poor water quality and high unionized ammonia (NH₃) levels [9]. Climate change is a major threat to fish productivity, and global warming has been considered one of the most important environmental problems affecting the world in the past 10 years [10]. Various aquatic species, including fish, plants, mammals, and corals, have suffered widespread mortality as a result [11].

According to Algammal et al. [12], bacterial infections have been identified as the main or the first cause of summer mass mortalities, posing a significant obstacle to tilapia fish production over the past 10 years. Stressed fish are susceptible to various bacterial pathogens [13]. Bacterial disease outbreaks lead fish farms to suffer significant financial losses due to their high mortality rates, particularly in the summer [14]. For aquaculture worldwide, disease and parasitism are the main causes of welfare, environmental, and economic concerns [15]. Furthermore, ecological elements such as salinity, pH, temperature, and oxygen level have been linked to disease spread, especially in intensive aquaculture systems [16–18].

**Figure 1.**
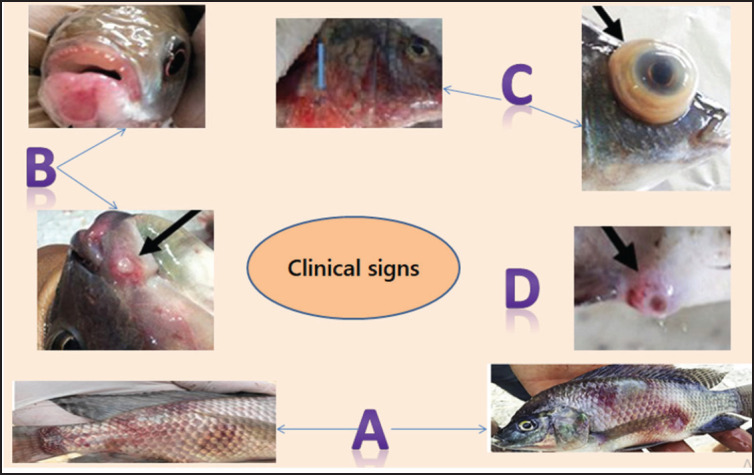
Clinical signs in Nile tilapia are affected by summer mortality syndrome: (A) red patches, (B) mouth ulcers, (C) exophthalmos, and (D) inflamed vent (Adapted from Ali et al. [19]).

Fish with bacterial infections [19] exhibit similar clinical signs, such as swimming aimlessly near the surface, reduced appetite, and various pathologic conditions such as tail rot, dermal ulceration, hemorrhagic skin and fins, exophthalmia, red sores, erected scales, and erythroderma (Fig. 1). Postmortem abnormalities associated with bacterial infections include conspicuous localized hemorrhages, spleen congestion, and necrosis in the liver and gills. *16S rDNA* sequencing provides accurate and detailed information, even for rare isolates [19–21].

Additionally, Choi et al. [22] reported that internal observations and diagnostic results revealed some reddish-brown liver, intestinal bleeding, and congestion in the fish’s abdominal cavity as clinical indicators of Korean rockfish. Swollen filaments in the gills were shown to result from mucus secretion and accumulation of foreign material. Some studies have found that hemorrhagic patches spread on the external body surfaces with fin rot in tilapia fish [7]. Internally, ascites was noticed with the presence of blood-tinged fluid in the abdominal cavity. The same authors found that the liver and spleen of tilapia fish with summer mortality syndrome were enlarged and friable.

Recently, there has been a significant increase in summertime Nile tilapia mortality rates in fish farms. Approximately 37% of fish farms in Egypt were affected by outbreaks of summer mortality syndrome, which had an average mortality rate of 9.2% and cost close to US$100 million [23]. The precise causes of mortality are unknown. However, certain risk factors such as the type of culture system, water quality, elevated temperatures, and increased salinity have been proposed to predispose fish to this mortality [24]. Ali et al. [19] reported that each of the predisposing causes contributed to the mass mortality event. Furthermore, according to other studies, the main cause of the common disease impacting Nile tilapia in farms may be bacterial infections such as *Aeromonas veronii* [13]. Environmental and interdisciplinary microbiological factors have also been identified as the main reasons for fish mass mortalities in other studies [25].

The most significant concern in fish culture systems is bacterial disease, which can lead to high mortality rates among farmed fish, especially in stressful conditions. According to Rathinam et al. [26], Gram-negative bacterial infections are the main cause of elevated mortality in freshwater fish farming. Most of these bacteria are *Aeromonas*, *Flavobacterium*, *Edwardsiella*, *Pseudomonas*, and* Vibrio*. Many studies have documented disease outbreaks in a variety of commercially significant fish species due to *Aeromonas *spp. infestations, including channel catfish [27], salmonids [28], Nile tilapia [29], pangasius catfish [30], common carp [31], koi carp [32], and silver carp [33], among others. Additionally, research has shown co-infections of *Aeromonas *spp. with epizootic ulcerative syndrome (EUS) [34], Cyprinid herpesvirus-2 [35], tilapia lake virus (TiLV) [36], and ich parasite [37] in fish culture systems.

In numerous freshwater fish farms, bacterial infections account for the majority of severe morbidities and fatalities among cultivated fish (Nile tilapia), and summertime temperatures can have a substantial impact on mortality rates [38]. Diseases of cultured fish negatively impact the attainment of optimum potential in fish farming. Bacterial infections cause 80% of fish mortality in tilapia production [39]. Two distinct biotypes of *Streptococcus agalactiae* were identified: biotype I (β-hemolytic) and biotype II (non-hemolytic). Biotype I causes acute mortality patterns [40], whereas biotype II causes chronic and persistent mortality patterns. After samples taken in the summer of 2017 were subjected to bacteriological analysis, 160 bacterial isolates were identified and classified as *Aeromonas hydrophila*, *Pseudomonas putida*, *Pseudomonas fluorescens*, and *Vibrio cholera* with the percentages of 50%, 15.62%, 21.87%, and 12.5%, respectively, while in the winter of 2018, 100 samples contained the following three microorganisms: *V. cholera* and *A. hydrophila*, with percentages of 50%, 37%, and 13%, respectively. In all, 40 samples were isolated in the summer of 2018; of them, 47.22% contained *A. hydrophila*, 22.22% contained *P. fluorescens*, 13.88% contained *P. putida*, and 16.66% contained *V. cholera*. According to the findings of a seasonal spread of bacterial strains, *A. hydrophila* has the highest mortality rate among naturally damaged farmed fish, and summertime mortality rates are higher than wintertime ones [38, 41].

One of the largest fish in the world that is only found in freshwater is the Murray cod (*Maccullochella peelii*), a well-known and valuable freshwater fish native to Australia. Due to their excellent nutritional value and rising economic significance, Murray cod have been successfully produced and raised recently, making them one of China’s most important new commercial fish. However, in farmed Murray cod, reports of bacterial and viral infections leading to disease epidemics have been made [42,43]. A new infectious disease has surfaced in China that has killed large numbers of young Murray cod. In the current study, fish with viral nervous necrosis showed aberrant swimming behaviors, including spinning close to the water’s surface, plunging to the bottom, and then rising to the surface again. The moribund fish also displayed clinical signs such as anorexia and skin discoloration. There were no noticeable gross pathological abnormalities seen during the necropsy [44]. According to a recent study by Magouz et al. [45], the most common bacterial infections found during mass-farmed tilapia summer mortality include* A*. *hydrophila*, *Vibrio *sp., and* Streptococcus iniae*. This finding supports the theory that warm water could increase the likelihood of these bacterial illnesses, leading to a large number of dead tilapia and significant financial losses. Recently, aquaculture has gained increasing significance as a key contributor to global food security, and Egypt has been at the forefront of advancements in fish farming. However, the aquaculture industry faces notable challenges, such as infectious diseases being a primary concern [59].

In this review, the primary objective is to highlight the key reasons for summer mortality syndrome in fish and discuss potential approaches to mitigate these negative effects. Additionally, we will explore the role of environmental conditions in this phenomenon.

## Summer Mortality Syndrome

The unidentified phenomenon affecting cultured farms of Nile tilapia (*Oreochromis niloticus*) was commonly called “summer mortality syndrome.” This disease occurred in the summer season and was characterized by a high mortality rate and a general symptom of septicemia in dead and moribund fish [19, 46].

Summer mortality syndrome was first documented in scientific literature by Fathi et al. [23], although it began affecting tilapia farms in 2012. The first reports of mass fatalities in the impacted marine fish farms were made in 2014; however, the spread in 2019 that hit the farms was particularly severe [47]. Mariculture farms have experienced annual losses due to disease outbreaks, especially during the summer months, known as summer mortality syndrome.

According to Ali et al. [48], every epidemic is distinguished by numerous general indications of septicemia in affected fish. Mortality of stocked fish ranged between 30% and 70%, with some instances reaching 100% [49]. Other research suggests that microbiological causes and interdisciplinary environmental factors were responsible for the mass fatalities [25].

Increasing production of aquaculture is necessary to meet the world’s food demands; fish diseases must be prevented and controlled, antibiotic use must be reduced or replaced, and environmental protection must be enhanced to ensure the safety of both human and animal health [50,51]. Immunostimulant factors play a crucial role in supporting aquaculture growth and survival by preventing opportunistic diseases using antioxidants and enhancing natural defense mechanisms [50,52]. *Aeromonas hydrophila* is one of the most common pathogenic bacteria associated with the cytokine responses in common carp [53]. Mass mortalities caused by *A. hydrophila* infections are frequently observed in aquaculture, resulting in substantial financial losses [54]. According to Choi et al. [22], the substantial temperature changes in the water due to typhoon Wukong during the summer of 2006 may have weakened the fish physiologically, resulting in mass mortality in Korea. Summer mortality has been reported in farmed species in Australia [55]. The complex interplay between biological, environmental, anthropogenic, and dietary stresses is likely to contribute to summer mortality [56]. Hooper et al. [57] suggest that these stressors can lead to physical and immunological impairments in abalone, increasing their susceptibility to opportunistic bacterial infections and eventual death. Reports indicate an increased presence of bacteria, primarily *Vibrio *spp., in abalone death incidents [56].

Recently, in the Arabian Gulf, Ibrahim et al. [58] identified that larval tapeworms (plerocercoid) can infect the Indian halibut (*Psettodes erumei*) with an overall prevalence of 15.4%. They reported that the seasonal prevalence was the highest in summer (14.6%), followed by spring (10.6%), winter (4.4%), and autumn (3.5%). Infection rates increased with fish size. Moreover, Abdelkhalek et al. [59] reported that summer conditions can cause many clinical signs, such as encysted metacercariae of digenetic trematodes, which displayed an array of clinical signs, including lethargy, erratic swimming behavior, loss of appetite, darkening of the skin, loss of scales, emaciation, stuck abdomen, tail and fin erosions, abdominal hydropsy, excessive mucus production, respiratory distress, and impaired growth in tilapia fish. From the above, it is clear that this phenomenon negatively affects fish production in several countries. Since it was mentioned in 2017 until now, it still threatens fish production during the summer, so we aim in this review to reveal more causes of infection as well as methods of prevention.

## Reasons for Mortality in Fish

**Figure 2.**
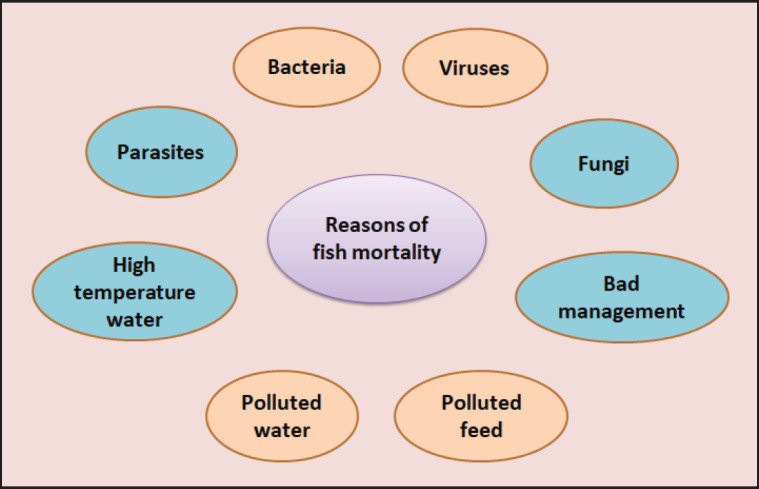
Reasons for summer mortality syndrome in fish. Environmental factors such as polluted water, high water temperatures, and contaminated feeds can induce the release of bacteria, viruses, and fungi, which can affect the health and well-being of fish and increase mortality rates in fish farms.

Aquaculture is one of the world’s most active and rapidly expanding food production industries, offering a potential solution to meet the global demand for aquaculture goods [26]. However, aquaculture faces numerous challenges, with diseases being the primary concern. Bacteria, viruses, parasites, fungi, and noninfectious factors all contribute to the global output deficit in aquaculture [58–60]. Fish diseases are complex and rarely result from a simple relationship between a virus, a host fish, and an environmental factor. The causes and routes of infection in fish are diverse, with additional issues such as low water quality further complicating the problem and increasing the occurrence of sickness [22, 23]. Many infections are common and feed on fish and saprophytes in soil, water, and invertebrate hosts such as crabs and snails. The majority of infections are caused by stress, and poor fish health management allows pathogens to come into direct and indirect contact with farmed fish, which makes disease transmission easier (Fig. 2). Aly [41] has identified factors typically involved in the mechanisms behind fish disease outbreaks, including contaminated water supplies, culture facilities, eggs or fish stocks, and environmental elements associated with fish culture (air, soil, ponds, equipment, and pollutants in feed). Future trends should concentrate on the development of pathogen-resistant fish strains or innovative diagnostic tools.

## Bacterial Infections

Bacteria cause numerous diseases and significant fish mortality rates in aquaculture. The majority of the microorganisms are caused by saprophytes, which are naturally occurring microorganisms that develop and reproduce by utilizing the organic and mineral matter substances in the aquatic environment. Summarized bacterial pathogens in fish mortality syndrome are presented in Table 1. Fish’s natural bacterial flora reflects the water’s bacterial population. Most of the bacteria that cause disease in fish are small, Gram-negative rods and members of Pseudomonadaceae, Vibrionaceae, and Enterobacteriaceae. Bacteria typically cause ulcerative and septicemic disease states. High mortality rates in fish stocks can also be brought on by the long myxobacteria (Gram-negative) of the family Cytophagaceae, which are not known to cause pathogens in warm-blooded animals.

Even though Gram-positive bacteria, some of which are even acid-fast, are less prevalent, under the right conditions, they can cause significant harm to some fish species. In Egypt studies, the most types of bacteria that caused mortality rates in many fish species were *Flexibacter columnaris*, *Flavobacterium columnare*, *P. fluorescens*, *Streptococcus faecalis*, Enterobacteriaceae, namely* Edwardsiella tarda*, and* Yersinia *spp., *P. fluorescens*, *S. iniae*, and *Streptococcus pneumoniae* [61–64].

Several Gram-negative bacteria, such as *A. hydrophila*, *A. veronii*, and *Aeromonas jandaei*, have been identified as pathogens that can cause ulcerative lesions and hemorrhages on the skin, liver, kidney, spleen, and swim bladder of cultured tilapia. These infections can also lead to conditions such as ascitic fluid accumulation, fatty liver, and enlarged hemopoietic tissue [65]. According to Dong et al. [66], a filamentous, Gram-negative bacterium called *F. columnare* is responsible for gill necrosis, fin erosion, and skin lesions. Concurrent infections with *A*. *veronii* and other opportunistic organisms have also been connected. According to Nguyen et al. [67], tilapia infected with *F. noatunensis* subsp. *orientalis* exhibits skin darkening, exophthalmia, irregular swimming, gill pallor, skin ulcers, and multifocal white. Additionally, *S. agalactiae* and *S. iniae*, two Gram-positive bacteria that cause severe mortality rates in fish when they exhibit clinical signs such as spiraling erratic swimming behavior, pop-eye, and hemorrhages, can infect tilapia [68]. Emerging as a potential zoonotic pathogen [69]. According to Vendrell et al. [70], *Lactococcus garvieae* has been linked to human cases in the USA, France, Canada, Taiwan, and Great Britain. The bacteria are Gram-positive and cause exophthalmia, internal lesions, lack of appetite, melanosis, lethargy, and hemorrhagic septicemia. According to Anshary et al. [71], it has been documented in tilapia fish from Indonesia, Brazil, and Thailand.

**Table 1.** Bacterial species responsible for fish mortality syndrome.

Species of fish affected	Species of bacteria	References
Catfish	*Aeromonas *spp.	Baumgartner et al. [27]
Freshwater fish	*Aeromonas*, *Flavobacterium*, *Edwardsiella,* and *Pseudomonas*	Rathinam et al. [26]
Salmonids, Channel catfish, Common carp, Nile tilapia, Pangasius catfish, Koi carp, Indian major carps	*Aeromonas *spp.	[28–33]
Nile tilapia	*Flavobacterium columnare*	Mohamed and Saleh [64]
Common carp, monosex Tilapia, and Nile tilapia,	*Yersinia ruckeri*	El-Gamal [61]
Grey mullet, Common carp, Nile tilapia, and African catfish	*Streptococcus faecalis*, *Edwardsiella tarda*, *Edwardsiella ictaluri*, Enterobacteriaceae (*E*. *tarda* and *Yersinia *spp.), *Aeromonas hydrophila* and *Pseudomonas fluorescens*	Abd El-Sattar [63]
Nile tilapia	*Streptococcus iniae* and *Enterococcus faecalis*	Anshary et al. [71]
Marine water fish	*Vibrio brasiliensis* and *Vibrio harveyi*	Montanchez and Kaberdin [72]
Nile tilapia	*Aeromonas *spp., *Streptococcus agalactiae*, *Staphylococcus *spp., *Vibrio *spp., *Plesiomonas shigelloides*, *Pseudomonas oryzihabitans,* and *Acinetobacter lwoffii*	Ali and Aboyadak [49]
Marine water fish	*Tenacibaculum maritimum*	Brosnahan et al. [73]
Nile tilapia	*Aeromonas hydrophila*, *Vibrio *sp*.*, and *Streptococcus iniae*	Magouz et al. [45]
Thin-lipped grey mullet (Liza ramada), European seabass (*Dicentrarchus labrax*), and gilthead seabream (*Sparus aurata*)	*Photobacterium damselae* Subspecies including *P*. *damselae *subspecies* damselae *and* P*. *damselae* subspecies *piscicida*	Eissa et al. [47]

Further research is required to fully comprehend the pathogenic impact of *Vibrio brasiliensis* on farmed animals. Additionally, *Vibrio harveyi* has been identified as a significant aquatic animal pathogen [72]. Ali and Aboyadak [49] reported that the most widespread pathogens were *Aeromonas *spp. (42%), *S. agalactiae* (14.5%), and *Vibrio *spp. (21%). Other evolving infections, such as *Staphylococcus *spp. (8%), *Plesiomonas shigelloides* (10%), *Acinetobacter lwoffii* (2.3%), and *Pseudomonas oryzihabitans* were also identified. In a recent study, Magouz et al. [45] reported on the identification and isolation of bacteria, including* Aeromonas* species, *Pseudomonas* species, *Vibrio *species, *Edwardsiella* species, *Streptococcus *species, and *Enterococcus* species. Recently, a study by Eissa et al. [47] aimed to uncover the underlying causes of massive fish kills and suggest an emergency control strategy in Egypt focusing on bacterial infection diseases. Moribund farmed thin-lipped grey mullet (*Liza ramada*), European seabass (*Dicentrarchus labrax*), and gilthead seabream (*Sparus aurata*) have demonstrated emaciation, congested gills and fins, ascites, skin darkness, skin erosions, and ulcerations. Internally, moribund fish emitted an unpleasant odor upon opening the abdomen, together with severe congestion and hemorrhages in the kidneys and brain. Mottled atrophied spleens were the most prominent findings, while the gastrointestinal tracts were filled with whitish caseous material. The liver was pale with multiple whitish nodules. *Photobacterium damselae* was the most retrievable bacterial pathogen from most infected fish and trash fish. *Photobacterium damselae *subspecies* damselae *and* P*.* damselae* subspecies *piscicida* were definitively identified from examined moribund fish using both conventional morpho-chemical and molecular assays. The authors suggested that this bacterial infection may be attributed to poor water quality, which was profoundly incriminated in triggering bacterial infections leading to mass mortality [47].

## Parasitic Infections

The most frequent cause of disorders in fish farms is parasites. Both obligatory and opportunistic parasites exist. Obligatory parasites require the host’s protection to survive and proliferate. Thus, many parasites that cause minor issues were discovered in wild fish. Problems can arise when diseased fish are introduced to a lab or an intensive culture system [76–79]. The fish are usually overcrowded, and the parasites that multiply are not separated as they would be in nature. Fish parasitic diseases are divided into three categories: helminthic, protozoan, and crustacean disorders. Protozoans can cause internal and external infections depending on their location, while most crustaceans are external parasites that cause harmful diseases. Most digeneans cause internal parasitic disorders, while annelids and monogeneans typically cause external parasitic ailments. Internal parasite diseases include infections by nematodes, cestodes, and acanthocephalans [80–85]. Due to the low host specificity of the adult stage, several parasites with larval stages in freshwater fish can infect humans. Table 2 summarizes the parasitic infections that cause fish mortality syndrome.

According to Peddinti et al. [74], 18 freshwater fish species from 10 families in Andhra Pradesh’s River Penna were screened for metazoan ectoparasites. Only 12 of the fish species had at least one parasitic species present. The mean intensity of infected fish ranged from 44.3 (*Oreochromis niloticus*) to 0.1 (*Glossogobius giuris*), with the prevalence record being 98.9% (*Wallago attu*) to 30% (*Salmostoma bacaila*). The dominance hierarchy was found to be Monogena > Copepoda > Isopod. *Wallago attu* was found to have the most diverse parasite community. The study also revealed that ectoparasites can more easily enter the skin and gills of carnivorous fish due to the lower number of scales on their bodies.

Fish-borne zoonotic trematodes are attracting global attention, with more than 18 million people reported to be infected by these zoonotic trematodes by the World Health Organization [75]. Heterophyid flukes are a significant concern.

Recently, in Egypt, a cross-sectional analysis of fish-borne zoonotic trematodes in freshwater fish in three geographical areas revealed an alarming threat by Mahdy et al. [76]. The authors identified them as *Haplorchis pumilio*, *Prohemistomum vivax*, and *Pygidiopsis genata* based on morphological traits. Quantitative real-time PCR was used to compare the immune response *in O*. *niloticus* infected with EMCs to uninfected fish [76]. The results showed higher levels of *TNF*-*alpha* and *IL-1β* in infected fish. The presence of adult flukes and EMCs caused significant histological changes in both experimentally infected pigeons and naturally infected fish, including inflammation and muscle necrosis in the digestive tracts.

Okunade et al. [77] reported that a parasite found in crustaceans infects certain types of cultured fish. A total of 484 *Clarias gariepinus* fish samples were randomly selected from fish farms located in three distinct agro-ecological zones within the state of Lagos, Nigeria. The *Argulus parasite*, a type of crustacean parasite, is present in the gills of fingerling fish during the rainy season. Its prevalence and degree of infection are approximately 1.05%. Despite the ideal temperature and L-DO levels that should favor parasites, the prevalence of *Argulus *was found to be low due to successful management techniques. The study conducted by Silva et al. [78] in the Quilombola zone of Maranhão State, Brazil, focused on the diversity of fish ectoparasitic species, gill modifications in *Hoplerythrinus unitaeniatus* (Characiformes: *Erythrinidae*) and *Cichlasoma bimaculatum* (Perciformes: *Cichlidae*), and the water quality for fishing. The researchers examined the mucous, body surface, and gills of fish specimens to identify ectoparasites. They found that three phyla, Platyhelminthes, Trematoda, and Arthropoda, accounted for approximately 30.95% of the prevalence of ectoparasites. Histological analysis revealed that 23.80% of specimens had mild tissue damage, 4.77% had moderate to severe tissue alteration, and 9.52% had severe and permanent lesions [78].

Tilapias can also be affected by facultative opportunistic parasite infections caused by water molds, such as those in the Saprolegniaceae family. According to Chauhan [86], they exhibit clinical symptoms of hemorrhagic skin lesions, tissue necrosis, edema, hemorrhaging, and cellular infiltration, which may result in subsequent bacterial infections. *Ichthyobodo necator* is a causal agent of ichthyobodosis, as stated by Senthamarai et al. [87]. Fish from both fresh and saltwater can become infected by this parasite, including ornamental fish. This parasite specifically damages the fish’s skin and gills, affecting salmonid fry and fingerlings. The skin of diseased fish appears steel grey, and they develop blue or grey mucous. *Trichodina* spp. parasites infect both freshwater and saltwater fish and are characterized by cilia. They are among the most common ectoparasites in both farmed and wild fish [87]. Sick fish show signs of weakness, inactivity, and loss of appetite. *Ichthyobodo* is a flagellate ectoparasite that becomes highly active at 38°C [88]. Recently, in the Arabian Gulf, Ibrahim et al. [58] identified that larval tapeworms (plerocercoids) can infect the Indian halibut (*Psettodes erumei*) with an overall prevalence of 15.4%. They reported that the seasonal prevalence was the highest in summer (14.6%), followed by spring (10.6%), winter (4.4%), and autumn (3.5%). Infection rates increased with fish size. Future research should focus on the impact of climate change on parasite prevalence or the development of resistance to anti-parasitic treatments.

**Table 2.** Effects of parasitic infections on fish mortality syndrome.

Species of fish affected	Species of parasitic	References
Freshwater fishes	*Wallago attu* and* Salmostoma bacaila*	Peddinti et al. [74]
*Clarias gariepinus* fish	*Crustacean* parasite	Okunade et al. [77]
Freshwater and marine water	*Trematoda*, and* Arthropoda*	Silva et al. [78]
Freshwater and marine water	*Dactylogyrus *Sp.	Borji et al. [79]
Common carp and big head	*Dactylogyrus minutus* and *Lernaea cyprinacea*	Nematollahi et al. [80]
Fresh water and marine water	*Trypanosoma *(blood parasites)	Hayes et al. [81]
Wild and cultured fishes	*Microsporidia*	Weiss et al. [82]
Freshwater and marine water	*Chilodonella*	Sadeghi et al. [83]
Cultured fish	*Argulus *sp.	Aalberg et al. [84]
Ornamental fish	*Spironucleus*	Lloyd et al. [85]
Red-belly tilapia (*Coptodon zillii*), Nile tilapia (*Oreochromis niloticus*), and African catfish (*Clarias gariepinus*)	Fish-borne zoonotic trematodes	Mahdy et al. [76]

## Fungi Infections

Fungi cause numerous economically significant diseases that affect teleosts. The most significant fungal pathogens belong to the Oomycetes family, such as *Achlya*, *Saprolegnia*, and *Branchiomyces*, which are commonly encountered during winter and are associated with stressors. Some of these fungi are parasitic and widely distributed in aquatic environments. Oomycetes can produce motile biflagellate spores, which can lead to infestation at any time. Freshwater and estuarine fish of all ages and species are susceptible to the widespread and highly contagious fungal disease known as Saprolegniasis [41]. Pang et al. [89] reported that 225 fungal species were responsible for infections in 193 animal species, resulting in 357 possible pairings of pathogenic fungi and marine animal hosts. Among the 193 animal species, the most reported hosts of fungal infections were found to be Chordata (100 species, 51.8%) and Arthropoda (68 species, 35.2%). Various factors contribute to fungal infections in fish. These factors can affect either the fungus or the fish, and an infestation is typically caused by a combination of factors rather than a single condition. Fungi causing Saprolegniasis have long been considered secondary infections. Lesions are often observed following handling, traumatic skin injuries, in crowded environments, pollution, or in conjunction with bacterial, viral, or parasitic diseases. Temperature is a significant factor influencing the growth of infestations, with most epizootic conditions occurring when temperatures drop below the ideal range for the fish species.

Numerous investigations have demonstrated that *Saprolegnia* spp. are pathogenic fungal species with thermal tolerance comparable to their host fish. They infect fertilized eggs, causing them to develop hairy tufts with a white cottony encapsulation, resulting in focal cytoplasmic infection and loss of cytoplasm. Senthamarai et al. [87] state that Oomycetes, a significant fungal group, are common in freshwater environments and cause fungal infections in both wild and farmed fish. Important aquaculture fungi include *Aphanomyces*, *Saprolegnia*, *Achlya*, *Calyptratheca*, *Pythiopsis*, *Thraustotheca*, *Leptolegnia*, and* Leptomitus*. These fungi thrive in fish exposed to environmental stressors such as low pH, L-DO, or high algal blooms. According to Choudhury et al. [90], common fungal illnesses in fish include Saprolegniasis, *Branchiomycosis*, EUS, and *Ichthyophoniasis. *Saprolegniasis is a common infection in freshwater and estuarine fish in warm equatorial environments. These opportunistic fungi induce immunosuppression by feeding saprophytically on decomposing organic matter. The development of *Saprolegnia *infection is greatly influenced by temperature, with outbreaks in some fish species occurring at physiologically low temperatures. *Branchiomyces demigrans* and *Brevibacterium sanguinis* are the main causes of branchiomycosis, a widespread issue in Taiwan and Europe.

## Viral Infections

Viruses are a significant cause of clinical and subclinical problems that impact the profitability of the fish farming sector. Viruses infecting fish species are not well understood, despite the presence of members of the 12th viral family worldwide in wild and farmed fish. A research system dedicated to studying fish viruses could help bridge this knowledge gap. By combining molecular biology technology, genetic markers, and multidisciplinary collaboration, a more comprehensive understanding of the fish virus situation could be achieved in the coming years [91,92].

The Tilapinevirus, which belongs to the Amnoonviridae family, contains the single-stranded RNA virus known as the TiLV, as identified by Thawornwattana et al. [91]. According to Tattiyapong et al. [92], the TiLV is a viral illness that spreads intercontinentally and causes significant mortality in fish. Authorities have ensured that all available information regarding the discovery of the Tilapia virus in tilapia fish is not confidential, despite conflicting claims about the presence of the TiLV in fish that died during the summer [23]. Many countries, including Malaysia, Bangladesh, Thailand, Ecuador, Indonesia, Uganda, the United States, and others, have reported widespread outbreaks of the tilapia virus [93]. The tilapia virus affects all stages of tilapia life, particularly during high temperatures in the summer [7]. According to Aich et al. [94], it is contagious and can spread through vertical and horizontal transmission routes. Fish infected with the tilapia virus experience significant damage to their liver, brain, and eyes. The virus causes highly pathogenic changes, including the formation of syncytial cells and severe hepatic necrosis with karyolysis and pyknotic nuclei in the hepatocytes [94]. Six virulence genes (*saga*, *scpI*, *simA*, *cpsD*, *pgm*, and *pdi*) were found to be involved in the pathogenesis of streptococcal infections in fish by Legario et al. [95] in some strains of *S. agalactiae* isolated from clinical infections and fatalities in cultured Nile tilapia fish. The pathogenicity of *S. agalactiae* was confirmed through an experimental infection of Nile tilapia fish. The high cumulative mortality and characteristic signs of streptococcosis exhibited by the challenged fish were like those reported by Abu-Elala et al. [96] and Sudpraseart et al. [97] and observed in naturally infected fish.

Several virulence genes have been identified in *S. agalactiae* isolates [98]. The histopathological findings of shrimp muscles affected by Loose Shell Syndrome showed sporadic hemocytic infiltration, which differed from the findings in *Litopenaeus vannamei* infected with infectious myonecrosis virus and the nodavirus PvNV, known to cause muscle necrosis [99]. Laem Singh virus was identified in a *Penaeus monodon* affected by Loose Shell Syndrome in a study on viral infections in shrimp [100]. The virus first emerged in 1992 and has since spread to various countries, including Indonesia. Although the TiLV is not zoonotic, its recent emergence in three continents—Africa, Asia, and South America—has sparked significant interest in tilapia fish health worldwide [101]. Since its initial discovery in Israel in 2014, TiLV has been identified in 13 countries, as reported in publications and government notifications to the World Health Organization (OIE). Various organizations, including the World Organization for Animal Health (OIE), the CGIAR Research Program on Fish Agri-food Systems, the FAO Global Information and Early Warning System, the Network of Aquaculture Centers in Asia-Pacific, and Jansen and Mohan [102], have all contributed to raising public awareness about the disease.

## Infectious Diseases in Fish Hatcheries

Most of the pathogens in fish hatcheries are bacteria and parasites. According to Faruk et al. [103], 76.66% of Bangladeshi hatcheries adopted equipment disinfection as a biosecurity precaution, possibly due to a failure to recognize its significance. Several fish-pathogenic Flavobacterium species, such as *Flavobacterium psychrophilum*, *Flavobacterium*, and *Flavobacterium branchiophilum*, belong to the family Flavobacteriaceae (phylum Bacteroidetes) and cause significant losses in farmed fish populations globally. Recent studies have shown that ovarian fluid and unfertilized eggs contain various species of *Flavobacterium* and *Chryseobacterium* [104–106]. The highest infection intensity (measured in counts per fish) was observed in silver carp (3–53), mirror carp (4–28), and grass carp (4–22) among immature carp. Among mature fish, silver carp (6–60) had the highest intensity, followed by grass carp (4–30) and mirror carp (10–20) [107]. According to Kyule–Muendo et al. [108], 76% of fish farmers reported mortality in their hatcheries and farms, resulting in an overall loss of almost 10%. However, these farmers did not attribute the mortality to fish infections, indicating that they considered them a common event.

## Environmental Effects

According to AlAsely et al. [39], unfavorable environmental factors significantly contribute to the spread of infections and the occurrence of mass fatalities. Climate change has a negative impact on fish products, especially due to global warming, where high temperatures, such as heat waves, can lead to heat stress and immediate physiological effects [109]. One of the major challenges in Atlantic salmon culture in sea cages is high water temperatures, as climate change is increasing average ocean temperatures and the frequency and severity of heat waves [110]. Recent reports have shown that high summer temperatures have adversely affected the production of sea-caged salmon in Tasmania [111], and 2.9 million salmon died in Newfoundland, Canada, in the summer of 2019 [112].

The anterior kidney of teleost fish contains steroidogenic cells that release cortisol, the primary glucocorticoid in fish. According to Martinez-Porchas et al. [113], heat stress activates the hypothalamus–pituitary internal axis, leading to the release of cortisol. Many fish species release cortisol in response to prolonged or severe exposure to high temperatures [114]. Reactive oxygen species generation induced by heat stress is the cause of oxidative damage in various biomolecules and tissues, as stated by Kamyab et al. [115].

The three most important oxidative stress indicators are catalase, malondialdehyde, and superoxide dismutase. They serve as an effective indicator for tracking the heat-induced oxidative stress response. Fish behavior, immunology, and physiology are all negatively impacted by L-DO, which is one of the most restricting elements in fish production. Fish immunological responses are directly impacted by hypoxia, which also heightens their disease susceptibility [116]. According to Cheng et al. [117], oxidative stress is triggered by intracellular reactive oxygen species, which are, in turn, triggered by toxic ammonia. Ammonia also disrupts intracellular calcium homeostasis, which damages DNA and causes cell death.

As water temperature increased, dissolved oxygen significantly decreased due to the negative correlation between oxygen solubility and temperature [118]. Fish exposure to chronic ammonia poisoning was measured by measuring hazardous ammonia levels in all the sea bass farms under study, which were 2–3 times higher than the permitted level. Analyzed farms that fed garbage fish had harmful ammonia levels that were directly elevated; lower dissolved oxygen levels also increased ammonia toxicity. According to Li et al. [119], persistent ammonia poisoning directly lowers the survival rate by causing fin, skin, and tail erosions as well as immune system suppression and high mortality.

Farm management, physical, and biological elements are subcategories of environmental factors, encompassing all farm biotic and abiotic components. By identifying these variables, a more focused approach to health management can be adopted, encompassing all choices that affect the disease’s expression (feeding, pond bottom management, stock density, and water quality management) [120]. Low oxygen levels worsen losses caused by the Summer Syndrome and have occasionally been observed in conjunction with significant mortality rates [121].

Exposure to environmental stressors can lead to changes in physiology, biochemistry, molecular, and DNA methylation patterns in fish, affecting global DNA methylation and disrupting biological pathways [122–123]. The majority of fish diseases are brought on by opportunistic bacterial pathogens that prey on stressed fish. Even in the absence of external stressors, certain illnesses can be rather serious. Gram-positive bacteria follow Gram-negative bacteria as the main offenders in these outbreaks. Pathogens such as *Vibrio *spp., *Photobacterium damselae *subsp*. piscicida*, *Renibacterium salmoninarum*, *Tenacibaculum maritimum*, *Edwardsiella *spp., *Pseudomonas *spp., *Streptococcus *spp., *Aeromonas *spp., *and Mycobacterium *spp. pose significant threats to fish in polluted aquatic environments. Effective management strategies and strict regulations are essential to prevent or minimize the impact of marine pollutants on aquatic animal health [123]. This strategy could assist regulatory authorities in developing effective management strategies to combat marine pollution, ensure the sustainability of commercial marine fisheries, and safeguard aquatic animal health.

## Utilizing Methods to Reduce the Causes of Fish Mortality

Fish mortality is a significant issue in aquaculture, often resulting from a complex interplay of factors including pathogenic infections, environmental stressors, and suboptimal management practices [1–5]. To effectively mitigate fish mortality, it is crucial to address these underlying causes through a range of preventive strategies. This section discusses methods that can be implemented to reduce the risk of fish mortality, focusing on two major areas: antibiotics and medicinal plants.

1. Antibiotics: managing pathogenic infections

One of the primary contributors to fish mortality is the outbreak of bacterial infections, which can rapidly spread and cause significant losses in aquaculture systems. The use of antibiotics has been a conventional method for controlling bacterial diseases in fish. Antibiotics can help eliminate pathogenic bacteria, preventing the spread of infection and reducing mortality rates [9–10].

However, the use of antibiotics must be carefully managed to avoid the development of antibiotic resistance, which is an existential threat to aquaculture and public health.

Key strategies for antibiotic use:

Targeted treatment: Using antibiotics specifically for diagnosed bacterial infections rather than prophylactic or blanket treatments.

Proper dosage and administration: Ensuring the correct dosage and duration of antibiotic treatment to maximize effectiveness and reduce the likelihood of resistance.

Integrated disease management: Combining antibiotic use with other methods, such as improved biosecurity practices and monitoring, to minimize reliance on antibiotics.

Despite its effectiveness, the overuse of antibiotics can lead to resistance, making it essential to develop alternative or complementary methods for managing fish health [50].

2. Medicinal plants: natural alternatives for disease prevention

In recent years, the use of medicinal plants and natural compounds in aquaculture has gained attention as a sustainable alternative to chemical treatments, including antibiotics. Many medicinal plants have demonstrated antimicrobial, anti-inflammatory, and immunostimulatory properties, which can help enhance the fish’s natural defenses against diseases and reduce mortality rates [100].

Prominent medicinal plants used in aquaculture:

Garlic (*Allium sativum*): Known for its antimicrobial and immunomodulatory properties, garlic can help combat bacterial infections and strengthen the immune response of fish [65].

Neem (*Azadirachta indica*): This plant has shown efficacy in controlling various parasitic infections and improving overall fish health [69].

Ginger (*Zingiber officinale*): With its anti-inflammatory and antioxidant effects, ginger is often used to boost fish immunity and prevent diseases [102].

By integrating medicinal plants into aquaculture practices, farmers can reduce their reliance on chemical treatments, promote sustainable practices, and decrease the risk of antimicrobial resistance.

3. Water management: environmental stressors and mortality prevention

Environmental stressors, such as changes in water quality, oxygen levels, and temperature fluctuations, can significantly impact fish health, leading to increased mortality [47]. Proper water management is essential for maintaining optimal conditions for fish growth and survival.

Key strategies for effective water management:

Maintaining optimal water temperature: Regularly monitoring and controlling water temperature to prevent thermal stress, which can lead to weakened immune responses and increased susceptibility to diseases [75].

Oxygenation: Ensuring adequate oxygen levels in the water, as low oxygen concentrations can stress fish and increase mortality [59].

Water filtration and treatment: Implementing effective filtration systems to remove toxins and pathogens, ensuring a clean and healthy environment for the fish [59].

Proper water quality management not only reduces stress-related mortality but also promotes overall fish welfare and growth.

4. Management practices: enhancing fish health through good practices

In addition to using antibiotics, medicinal plants, and improving water quality, good management practices play a pivotal role in reducing mortality rates in fish farming. Implementing effective biosecurity measures, proper feeding regimes, and regular health monitoring can significantly improve fish survival rates [28].

Best management practices include:

Routine health monitoring: Regular health checks and early detection of diseases can prevent outbreaks and minimize mortality [75].

Biosecurity measures: Ensuring clean tanks, equipment, and personnel to prevent the introduction and spread of pathogens [95].

Optimized feeding practices: Providing a balanced diet that meets the nutritional needs of the fish, helping to maintain strong immune systems and promote overall health [25].

By integrating these management practices, fish farmers can create a healthier and more sustainable farming environment, reducing the risk of mortality.

## Controlled Mortality in Fish by Medicinal Plants

**Figure 3.**
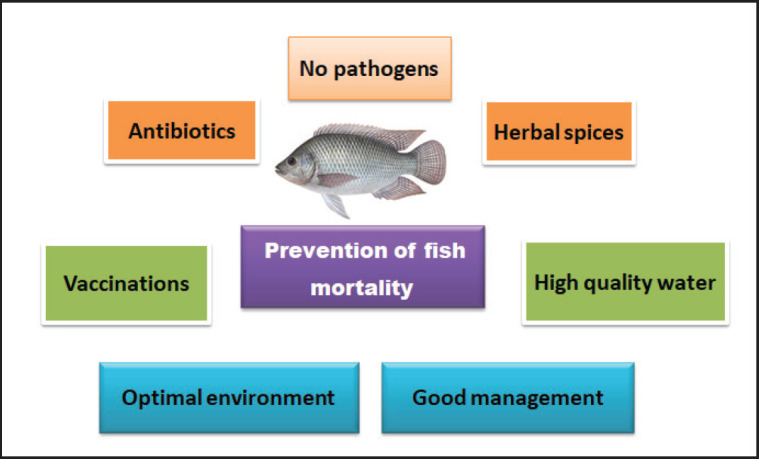
Prevention and control of fish mortality syndrome.

Abdel‐Tawwab et al. [124] found that incorporating green tea into experimental diets for Nile tilapia can enhance growth, feed consumption, and resistance to A. hydrophila infection, thereby improving fish health. The optimal level of green tea inclusion was determined to be 0.5 gm/kg of diet. In a study by Enany et al. [38], 100 Nile tilapia fish with clinical infections were randomly sampled from a farm in Egypt to identify the bacterial pathogens affecting the fish. Samples were collected from the brain, kidney, spleen, liver, and ulcers. Methods for preventing and controlling fish mortality are illustrated in Figure 3.

Medicinal herbs have been used as immunostimulants to enhance animals’ resistance to infection due to their ability to activate white blood cells [60]. The active components of plants, such as quinones, polyphenolics, terpenoids, alkaloids, and polypeptides, can effectively replace synthetic substances, antibiotics, and other medications, making them safe for both humans and the environment. These plant-derived products can promote growth and reduce stress, particularly in shrimp and finfish. Therapeutic plants include seaweeds, spices, herbs, herbal extracts, traditional Chinese medicines, and commercial plant-derived products [60].

Studies have shown that medicinal plants like *Withania somnifera*, also known as Indian ginseng, can reduce the mortality of Greasy Groupers (*Epinephelus tauvina*) infected with *V. harveyi* [125]. Fish fed with dietary ginger exhibited increased nonspecific immunity, leading to reduced susceptibility to *V*. *harveyi* infection. *Allium sativum*, the plant used in garlic, possesses immunostimulant, therapeutic, and antibacterial properties that enhance immunity against *V*. *harveyi*, promoting better growth and survival [126]. Similarly, Van Hai [60] discovered that feeding seaweed extracts to *Fenneropenaeus indicus* Indian prawns prevented them from contracting *Vibrio parahaemolyticus* infection.

One mangrove species that has been studied and is being developed as a replacement for other components in disease control, including the prevention of the White Spot Syndrome Virus in shrimp, is *Sonneratia alba* [127] in Indonesia. This type of mangrove grows in various locations near rivers and aquaculture sites. It is considered a potential substitute for disease prevention in tiger shrimp: *S. alba* [128–129]. This mangrove species has been identified as a potent antibacterial agent in shrimp, with minimum inhibitory concentrations of 1 mg/l for *V. harveyi* and 0.1 mg/l for *V. parahaemolyticus*, respectively. Other mangrove species that have shown the ability to inactivate the White Spot Syndrome Virus include *Rhizophora mucronata*, *Sonneratia *sp., and* Ceriops tagal*. The potential of *Sonneratia caseolaris* mangrove extract as an immunological stimulant has also been recognized. This type of mangrove can be used as an immune stimulant material in shrimp farming, as the methanol extract from it can enhance the immune response, phagocytic characteristics, and phenoloxidase activity in shrimp [127]. The use of methanol, ethanol, butanol, and other chemical solvents for mangrove extraction has been the subject of numerous investigations.

According to Muliani et al. [130], the butanol extract of *Bruguiera gymnorrhiza* and the diethyl ether and methanol extract of *S. alba* exhibit potent antibacterial properties. Both types of mangroves are believed to have anti-White Spot Syndrome Virus properties in addition to their antibacterial effects. Susianingsih [131] suggests that the survival rates of tiger shrimp can reach 100% when using methanol, butanol, and diethylene extracts from *S. alba* and *B. gymnorrhiza* instead of the control group. A recent study by Muliani et al. [127] indicates that tiger shrimp mortality can be reduced by using a 1% concentration of *S. alba* extract, which is most effective in inactivating WSSV.

## Controlled Mortality in Fish by Antibiotics

*Aeromonas hydrophila*, *P. putida*, *P. fluorescens*, and* V. cholerae *were identified as isolated fish pathogens based on morphological features, antibiotic sensitivity testing, and biochemical analyses. The results of the microbial sensitivity testing for the isolated strains revealed that the preferred antibiotics for *A*. *hydrophila* were tetracycline (TE30), tobramycin (TOB10), oxytetracycline (T30), norfloxacin (NOR10), and sulfamethoxazole–trimethoprim (SXT25). For *P. fluorescence*, the preferred antibiotics were tetracycline (TE30), tobramycin (TOB10), oxytetracycline (T30), and kanamycin (K30). For *P. putida*, the preferred antibiotics were tetracycline (TE30), nalidixic acid (NA30), streptomycin (S10), and kanamycin (K30). In contrast, the preferred antibiotics for treating *V. cholerae* were nalidixic acid (NA30) and oxytetracycline (T30). The results of the PCR test confirmed the findings of the antibiotic sensitivity test and the presence of *V. cholerae*, *P*. *fluorescence*, and *A*. *hydrophila *in the affected fish [127]. According to Matern [120], unfavorable environmental factors play a significant role in the spread of infestation and high-mortality outbreaks. Therefore, it is essential to maintain a cultured fish environment and proper husbandry practices to produce healthy fish. Assar et al. [132] demonstrated that phytochemicals could be a suitable substitute for controlling this infestation. Further research is needed to investigate the effects of olive leaf extract on the immune response and growth performance of common carp to determine the most effective way to use this extract as an immune stimulant for these fish.

## Prevention of Fish Disease

To enhance the tilapia-rearing environment and reduce pathogen virulence, it is essential to maintain optimal fish culture practices, environment, and husbandry procedures to ensure the health of the fish [45]. Prevention is a key aspect of any public health protection program, and it can be as challenging and complex as disease control efforts. Fish disease control encompasses both preventive and therapeutic measures. Preventive measures for fish diseases include various factors, such as regulatory compliance, nutrition and feeding practices, and genetic resistance to diseases. The following methods play a crucial role in disease prevention.

## Vaccination

The development of immunization techniques and research on fish immune responses has progressed swiftly [133]. While vaccinations cannot completely protect fish from infestation, they can assist them in fighting off infections to the extent that they are cost-effective in many situations where certain diseases lead to serious difficulties. Eliminating and de-infesting purging programs aim to stop fish infections from spreading from one area to another. The methods for ensuring the purging of fish production facilities, the disinfestation procedures, and the evaluation of the cost-effectiveness of different purging strategies have not been extensively documented in the literature [134]. The goal of egg disinfestation warfare is to stop infections from spreading horizontally from the egg facility to the keeping facility and vertically from the parent stock to the progeny. Purging techniques can be beneficial in keeping distinct fish populations apart while the fish are being raised. When a facility cannot be depopulated and disinfested in a single operation, phased disinfestations can be carried out. Completely disinfecting fish kills them, but it is simpler to do so. Because there is less contamination, complete disinfection offers a higher chance of success than a staged approach [134].

Since vaccination is the best immunological preventive approach against diseases, it has the potential to improve fish survival and enhance the profitability of [135,136]. The development of fish vaccines has garnered significant interest in studies focusing on vaccine composition, immunization schedules, and protective efficacy. Over time, fish disease diagnosis and immunization techniques have improved, making them more applicable to the aquaculture industry. Initially, fish vaccinations were limited to salmonid species before becoming widely adopted in aquaculture. The success of salmon farming can be largely attributed to vaccination, which is essential for large-scale commercial fish farming [137]. The advancements in salmonid farming due to vaccination have led to increased interest in cultivating other marine fish species [138]. Research and development efforts on vaccines for fish species have also been extended to Asian fish species, with commercial vaccinations now available for seabasses (*Lates* spp.), tilapias (*Oreochromis* spp.), and other species in Southeast Asian countries, as well as amberjacks (*Seriola* spp.) in Japan and grass carp (*Ctenopharyngodon idella*) in China [139]. Grouper species (*Epinephelus* spp.) have been the focus of numerous studies utilizing vaccination techniques to combat infections. Huang et al. [140] successfully inoculated orange-spotted grouper against pathogenic *S. iniae* using an intraperitoneal injection of a formalin-inactivated vaccine, resulting in a 100% survival rate 6 months post-inoculation.

## Green Water Technique

According to Defoirdt et al. [141], the larvae are cultivated in water rich in microalgae using the relatively new green water technology. Studies have indicated that the growth of *V. harveyi* in giant tiger prawns was effectively prevented by green water comprising eight bacterial isolates and 12 fungal isolates [142]. However, fish farms have not yet made extensive use of this approach. Aquaculture utilizes green water technologies to manage the growing environment [143]. In the rearing ponds employed for this method, fish larvae are surrounded by an abundance of microalgae, bacteria, and zooplankton. If the system water has been pretreated to remove competing bacteria, cultivated microalgae strains can be added to culture tanks, or the technique can be based on naturally occurring microalgal populations that are encouraged to grow with the addition of fertilizer. Better direct and indirect feeding of larvae, reduced stress levels, improved environmental conditions for feeding by increasing turbidity and light, and enhancing visual contrast, increased oxygenation rates, and increased antibacterial properties in rearing ponds are all considered major contributors to the better growth and survival rates reported by several authors [144]. The profitable and advantageous benefits of green water are linked to many processes, including the synthesis of bioactive chemicals by algal cells, which have antioxidant and antibacterial properties that regulate virulence genes. The most often utilized microalgal species for this purpose include *Chlorella*, *Nannochloropsis gaditana*, *Nannochloropsis *sp., *Isochrysis galbana*, *Isochrysis *sp., *and Tetraselmis *sp.

## Molecular Technology

**Figure 4.**
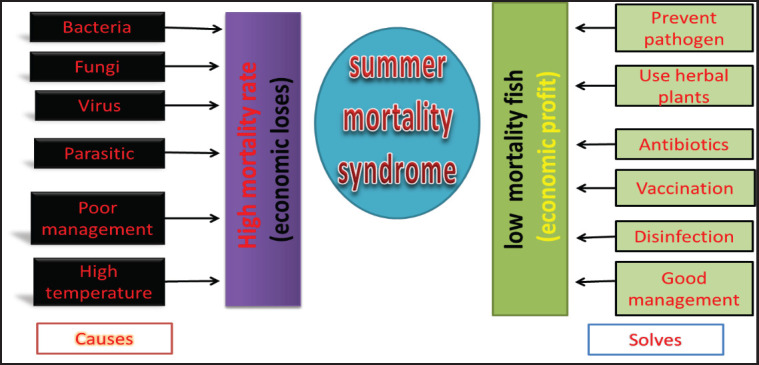
Summary of summer mortality syndrome in fish.

Thanks to the advantages of molecular innovation, fish genome sequencing can facilitate gene editing using recently developed genome editing technologies such as zinc-finger nuclease (ZFN), transcription activator-like effector nuclease (TALEN), and clustered regularly interspaced short palindromic repeats associated with Cas9 (CRISPR/Cas9). Studies on fish gene editing have already been conducted to demonstrate the viability and use of these methods. For example, sterile channel catfish were created using the ZFN technique to delete the pituitary LH hormone *β subunit* gene. In another study, common carp was modified using CRISPR/Cas9 to alter the mstnba gene, which codes for myostatin, an inhibitor of skeletal muscle growth, resulting in increased muscle growth. TALEN technology was used to disrupt the signal transducers and activators of transcription (STAT) gene, creating mutant zebrafish with scoliosis, a fractured spine, and deformed bone joints, highlighting the importance of STAT genes in vertebrate cell proliferation and differentiation. Transgenesis is another method used to create genetically altered tilapia resistant to illness. Fish with the growth hormone gene inserted into them are known as transgenic fish in the salmon industry. Transgenic fish showed a growth rate two to six times faster and a higher feed conversion rate compared to control fish. These findings suggest that introducing disease-resistant genes into tilapia to increase production may be a viable strategy for disease control in aquatic animals. According to a recent study, nanotechnology is a cutting-edge technology that is garnering interest worldwide due to the essential role of nanoparticles in various fields of science and technology. Different nanoparticles, such as zinc, selenium, silver, iron, and copper, have been utilized in the aquaculture industry to address various issues, including disease management, vaccine delivery, growth factor delivery, water cleaning, and nutrition delivery (Fig. 4).

## Conclusion

Aquaculture is one of the world’s most active and rapidly expanding food production industries. Fish not only serve as a major food source for humans but are also economically crucial, contributing to both human economic stability and nutritional well-being. Diseases pose the biggest obstacle to aquaculture and its global output. Summer mortality syndrome in fish can be caused by various factors, including bacterial pathogens, poor water quality, viral infections, parasitic infections, fungal infections, and compromised immune systems in fish. Fish can become more vulnerable to secondary illnesses as a result of poor water quality and management techniques. Methods for controlling and preventing fish diseases include the use of antibiotics (such as oxytetracycline, nalidixic acid, norfloxacin, sulfa-trimethoprim, tetracycline, and tobramycin), herbal medicine (such as green tea and olive leaf extract), improving environmental conditions, and implementing excellent management practices. Prevention of fish disease can be done by using many methods, such as vaccination, green water technique, and molecular technology. Further research is necessary to develop effective solutions to prevent disease outbreaks, maintain fish production, and support fish farmers. There are important reasons for continued innovation in fish farming practices to combat summer mortality syndrome effectively. Aquaculture is one of the world’s most active and rapidly expanding food production industries. Fish, in addition to being a major food source for humans, is also crucial economically, contributing to human economic and nutritional well-being. Diseases pose the primary obstacle to aquaculture and its global output. Summer mortality syndrome in fish can be caused by various factors, including bacterial pathogens, poor water quality, viral infections, parasitic infections, fungal infections, and compromised immune systems. Poor water quality and management practices can make fish more susceptible to secondary infections. Methods for controlling and preventing fish diseases include the use of antibiotics (such as oxytetracycline, nalidixic acid, norfloxacin, sulpha-trimethoprim, tetracycline, and tobramycin), herbal medicine (such as green tea and olive leaf extract), improving environmental conditions, implementing good management practices, and adopting innovative approaches such as vaccination, the green water technique, and molecular technologies such as CRISPR/Cas9. These advanced methods hold promise for improving disease prevention and boosting fish production. Further research is needed to develop effective, sustainable solutions to prevent disease outbreaks, maintain fish production, and support fish farmers worldwide.

## List of abbreviations

A, Aeromonas; EMCs, embryonic muscle cells; WSSV, white spot syndrome virus; PCR, polymerase chain reaction L-DO, low dissolved oxygen; TALEN, transcription activator–like effector nuclease; TiLV, Tilapia virus; V, Vibrio; ZFN, zinc-finger nuclease.
